# Nucleus accumbens deep-brain stimulation efficacy in ACTH-pretreated rats: alterations in mitochondrial function relate to antidepressant-like effects

**DOI:** 10.1038/tp.2016.84

**Published:** 2016-06-21

**Authors:** Y Kim, S McGee, J K Czeczor, A J Walker, R P Kale, A Z Kouzani, K Walder, M Berk, S J Tye

**Affiliations:** 1 School of Psychology, Faculty of Health, Deakin University, Melbourne, VIC, Australia; 2 Department of Psychiatry and Psychology, Mayo Clinic, Rochester, MN, USA; 3 Centre for Molecular and Medical Research, School of Medicine, Faculty of Health, Deakin University, Melbourne, VIC, Australia; 4 Metabolism and Inflammation Program, Baker IDI Heart and Diabetes Institute, Melbourne, VIC, Australia; 5 School of Engineering, Faculty of Science Engineering and Built Environment, Deakin University, Geelong, VIC, Australia; 6 Deakin University IMPACT Strategic Research Centre, School of Medicine, Faculty of Health, Geelong, VIC, Australia; 7 Department of Molecular Pharmacology and Experimental Therapeutics, Mayo Clinic, Rochester, MN, USA; 8 Department of Psychiatry, University of Minnesota, Rochester, MN, USA

## Abstract

Mitochondrial dysfunction has a critical role in the pathophysiology of mood disorders and treatment response. To investigate this, we established an animal model exhibiting a state of antidepressant treatment resistance in male Wistar rats using 21 days of adrenocorticotropic hormone (ACTH) administration (100 μg per day). First, the effect of ACTH treatment on the efficacy of imipramine (10 mg kg^−1^) was investigated alongside its effect on the prefrontal cortex (PFC) mitochondrial function. Second, we examined the mood-regulatory actions of chronic (7 day) high-frequency nucleus accumbens (NAc) deep-brain stimulation (DBS; 130 Hz, 100 μA, 90 μS) and concomitant PFC mitochondrial function. Antidepressant-like responses were assessed in the open field test (OFT) and forced swim test (FST) for both conditions. ACTH pretreatment prevented imipramine-mediated improvement in mobility during the FST (*P*<0.05). NAc DBS effectively improved FST mobility in ACTH-treated animals (*P*<0.05). No improvement in mobility was observed for sham control animals (*P*>0.05). Analyses of PFC mitochondrial function revealed that ACTH-treated animals had decreased capacity for adenosine triphosphate production compared with controls. In contrast, ACTH animals following NAc DBS demonstrated greater mitochondrial function relative to controls. Interestingly, a proportion (30%) of the ACTH-treated animals exhibited heightened locomotor activity in the OFT and exaggerated escape behaviors during the FST, together with general hyperactivity in their home-cage settings. More importantly, the induction of this mania-like phenotype was accompanied by overcompensative increased mitochondrial respiration. Manifestation of a DBS-induced mania-like phenotype in imipramine-resistant animals highlights the potential use of this model in elucidating mechanisms of mood dysregulation.

## Introduction

Symptomatically, depressive periods are characterized by decreased energy, paralleled by reduced brain energy generation.^1, 2^ This is highlighted by clinical studies examining glucose utilization, blood flow and energy metabolites in relevant depressive cohorts.^3, 4, 5^ Mitochondrial function has a central role in energy regulation, where it is directly influenced by glucocorticoids, oxidative stress, inflammation and antidepressants.^6, 7, 8, 9^ In turn, mitochondrial function regulates not only catabolic processes, but also production of free radicals and apoptotic processes for cellular homeostasis.^10^ Mitochondrial dysfunction may, therefore, lead to disruption of adaptive neural plasticity and an imbalanced cellular response to stressors. Notably, efficacious pharmacotherapies, including antidepressants, lithium and electroconvulsive therapy, seemingly affect mitochondrial function.^11, 12, 13, 14^ One of the most important functions of mitochondria is the generation of adenosine triphosphate (ATP), the molecular unit of intracellular energy, which powers numerous cellular processes and is needed to recover from insults including oxidative and nitrosative stress. This is essential for the maintenance of the above-mentioned processes including cellular integrity and neural plasticity.

The link between major depressive disorder, bipolar disorder and mitochondrial dysfunction has been under-recognized. The brain, as an intensely energy-dependent tissue, is particularly vulnerable to the effects of mitochondrial dysfunction—primary mitochondrial disorders are evidenced by neurological symptoms.^6^ A small but growing body of preclinical and clinical research, including proteomic analysis,^15, 16^ mitochondrial DNA data^17^ and imaging techniques,^5^ support a role for mitochondria in neuroprogressive processes that may occur in treatment-resistant depression (TRD).

The heterogeneity of major depressive disorder and the lack of animal models displaying a treatment-resistance phenotype is a challenge.^18^ Our preliminary model of TRD^19, 20^ includes typical physiological characteristics of the clinical major depressive disorder population, namely disturbances in the hypothalamus–pituitary–adrenal axis, brain mitochondrial function, dopamine transmission and antidepressant resistance. The nucleus accumbens (NAc), a key dopaminergic terminal region of the limbic system, is a neurosurgical target for antidepressant modulation.^21, 22^ In our earlier work, NAc deep-brain stimulation (DBS) has been shown to increase dopamine efflux in animals resistant to imipramine.^23, 24, 25, 26^ Direct electrical stimulation of the NAc region altered depression-related behaviors and restored an antidepressant response.^21, 24, 27, 28, 29^ Leveraging off this, investigation of the antidepressant and mania-inducing effects of NAc DBS in relevant animal models with face validity at the levels of neuronal circuitry, biochemistry and phenomenology has the potential to increase our understanding of the role of the underlying neurobiology, particularly mitochondrial function, in antidepressant treatment response.

In this study, we used chronic adrenocorticotropic hormone (ACTH) treatment to investigate the efficacy of NAc DBS in a cohort of tricyclic antidepressant-resistant animals with the behavioral changes assessed using the forced swim test (FST) and open field test (OFT). The aim of this study was to investigate mitochondrial function in an animal model with face validity and its potential association with treatment efficacy.

## Materials and methods

All experiments were conducted in accordance with the NIH guidelines for the care and use of laboratory animals and were approved by the Institutional Animal Care and Use Committee.

### Animals

This study utilized male albino Wistar rats weighing 200–400 g. Animals were housed individually, in controlled temperatures (20–22 °C) on a 12-h light–dark cycle. Food and water were available *ad libitum*. Animals entered the study at ~6 weeks of age following a week-long acclimatization period, and completed testing in their tenth week, at which point they were killed. Animals were randomly assigned into one of eight groups, under two conditions ‘Vehicle' and ‘ACTH' using simple randomization. Group sizes were justified using software G*Power 3.1 (Dusseldorf, Germany)^30^ for an estimated medium effect, with a power of 0.95. Control (CTRL) groups under each condition had *n* of 10, whereas surgery groups sized *n*=15 to account for potential ‘drop-outs'. Some animals had to be removed over the course of study for health reasons (for example, head-cap failure) and were thus removed from the analysis. As a result, final group numbers at the end of phase 2 were as follows: for ‘Vehicle (*N*=41)' condition including CTRL (*n*=10), SURG (*n*=12), SHAM (*n*=10), DBS (*n*=9); ‘ACTH (*N*=44)' including CTRL (*n*=10), SURG (*n*=11), SHAM (*n*=9), DBS (*n*=14). All procedures were carried out in accordance with the National Institute of Health Guide for care and use of laboratory animals (NIH publications no. 80-23) and were approved by the Institutional Animal Care and Use Committee of the Mayo Clinic.

### Drugs

The drugs used in this experiment included the following: isoflourane (~1.5%); ACTH 1–24 (AnaSpec, San Jose, CA, USA), 100 μg per day dissolved in distilled water; imipramine hydrochloride (Sigma-Aldrich, St Louis, MO, USA), 10 mg kg^−1^ dissolved in 0.9% saline; control vehicle 0.9% saline (Fisher Healthcare, Hanover Park, IL, USA); and FatalPlus (Vortech Pharmaceuticals, Dearborn, MI, USA; constituents: pentobarbital sodium 390 mg ml^−1^; propylene glycol 0.01 mg ml^−1^; ethyl alcohol 0.29 mg ml^−1^; benzyl alcohol (preservative) 0.20 mg ml^−1^) 0.70 cc. Drugs were administered via intraperitoneal injection apart from isoflurane, which was delivered via inhalation.

### Anesthesia and intraoperative monitoring

The animals were anesthetized with isoflurane inhalation in an induction chamber and were maintained with a nose cone (World Precision Instruments, Sarasota, FL, USA). The concentration of isoflurane ranged between 1 and 3% to ensure adequate sedation of the rat during surgery. Once anesthetized, the animal was placed in a stereotaxic frame (Model 1430, David Kopf Instruments, Tujunga, CA, USA). The skull was secured with a nose clamp, incisor bar and ear bars. Constant body temperature (36.5 °C) was maintained using a heat pad, and the animal's body temperature was measured using a digital thermometer placed under the abdomen. While under general anesthesia, the animal was monitored by respiratory rate and hind-paw pinch.

### Stereotactic surgery

A midline 1.5–2-cm incision was made starting just caudal to the eyes and ending just rostral to the ears to expose the two skull landmarks: bregma and lambda. A 2-mm diameter trephine hole was broached in the skull at the site corresponding to the targets. The dura matter was opened using a fine needle. Then, stimulating electrodes (twisted bipolar platinum iridum electrodes, 10 mm long, 0.075 mm in diameter; Plastics One, Roanoke, VA, USA) were placed bilaterally in the NAc core targets (anteroposterior +1.5, medial-lateral ±1.5, dorsoventral −7.0 from the bregma) using stereotactic coordinates.^31^

The electrodes were secured in place using dental cement. Four small jeweler screws were also placed in the skull surface to prevent sliding of the dental cement over the surface of the skull during the postoperative period.

### DBS

The electrodes were connected to a back-mounted DBS device and the battery connected (both the device and the battery were secured in a rodent jacket (Harvard Apparatus, Holliston, MA, USA)), so that the animals could not disturb the device. Animals remained awake throughout the course of DBS and were closely observed for the first hour of stimulation. We had not previously observed any adverse effects of DBS of the NAc using the currents applied in this study (130 Hz, 90 μS, 100 μA). DBS was continuously delivered for 7 days via the back-mounted DBS device. This occurred in the animal's home cage, and the animal was free to move about the cage without any restriction.

### Behavioral testing

#### OFT

The arenas used were 622.3 mm × 622.3 mm (CleverSys, Reston, VA, USA). Animals were each placed in the central zone of the arena, and were allowed to move freely for 6 min. Their behavior was recorded with a video camera. Data were then analyzed ‘blind' using the behavioral analysis package CleverSys TopScan. OFT was used to quantify locomotor, anxious and exploratory activities by observing the animals.

#### Porsolt FST

The forced swim apparatus (CleverSys) was used in this experiment (dimensions: 600 mm height × 200 mm diameter) and filled with tap water (25±1 °C) to a depth of 25 cm. Animals were exposed to a 15-min learning trial, conducted 2 h after the OFT. A 6-min test session was then conducted on the subsequent day. Sessions were recorded with a video camera, and then analyzed ‘blind' using the behavioral analysis package CleverSys ForcedSwimScan CleverSys) and validated with hand-scoring. The behaviors of interest included ‘*immobility*' (passive coping behavior), ‘*swimming'* and ‘*climbing'* (active coping behavior).

### Experimental procedure

**Figure 1:**
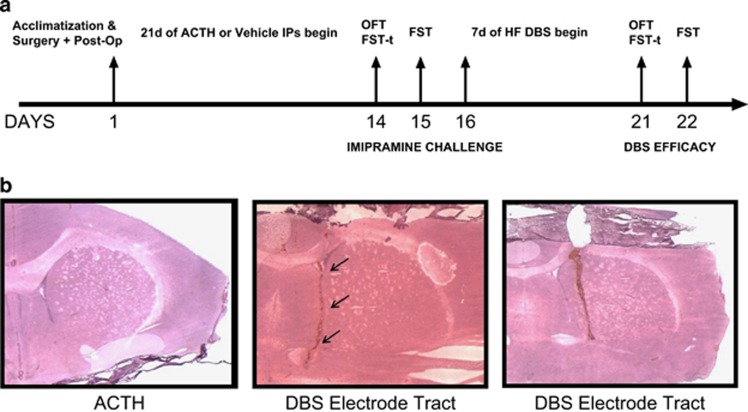
(**a**) A detailed outline of experimental timeline. (**b**) Hematoxylin and eosin staining validation of electrode tracts in deep-brain stimulation (DBS) animals treated with adrenocorticotropic hormone (ACTH). FST, forced swim test; OFT, open field test.

After 7 days of acclimatization, electrodes were implanted bilaterally into the NAc using stereotactic surgical procedures. Following recovery from surgery (3 days), animals received 14 days of injections of either saline (0.9%) or ACTH-(1–24; 100 μg per day). In order to test for antidepressant efficacy in the ACTH model, animals were challenged with imipramine (10 mg kg^−1^) on the 14th day for the OFT. Two hours post test, animals received their initial forced swim stress exposure for 15 min. Then, a FST was conducted on day 15 (6 min), 30 min after the second imipramine administration (Figure 1a).

DBS animals received high-frequency NAc DBS (130 Hz, 100 μA, 90 μs) after 14 days of ACTH or saline treatment, using a back-mounted DBS device. Behavioral tests were recorded for subsequent analyses. Final OFT, FST training and FST were conducted on days 21 and 22 to investigate the efficacy of DBS (Figure 1a). Throughout the course of DBS, stimulation was only disrupted during the 6 min of OFT and 15 min+recovery time (~30 min) of FST training to sustain the potential antidepressant effects of DBS. Animals were killed 30 min after FST, and their brains were harvested and cardiac blood samples collected. Isolated plasma aliquots of cardiac blood (centrifuged at 3300 *g* for 10 min) were taken and stored at −80 °C.

### Killing and dissection

Rats were killed with an intraperitoneal overdose of pentobarbital (20 mg kg^−1^). The brain was then dissected out of the skull. The prefrontal cortex (PFC) was removed and prepared for mitochondrial isolation. The remaining portion of the brain was frozen by placing it on dry ice, followed by storage at −80 °C.

### Immunohistochemistry

The remaining portion of the brain was embedded using Cryo-M-Bed (A-M Systems 527738). Six-milimeter coronal plane sections were taken of the NAc. The sections were fixed using ice-cold acetone for 10 min, followed by 10 min of drying time.

#### Hematoxylin and eosin staining

After fixation and drying, the slides were incubated in phosphate-buffered saline for 10 min, followed by 30-s incubation in hematoxylin stain, and then rinsed under running water for 5 min. The following dips were carried out: Blueing 4 dips, water 10 dips, water 10 dips, 95% Ethanol 4 dips, Eosin 6 dips, 50% Ethanol 12 dips, 70% Ethanol 12 dips, 95% Ethanol 12 dips, Absolute Ethanol 12 dips and Xylene 12 dips. Slides were allowed to completely dry before preserving in Vecta-mount (Vector Laboratories, Burlingame, CA, USA) and coverslipping. Refer to Figure 1b for electrode tract images.

### Freezing of tissue for mitochondrial isolation

The PFC tissue was removed immediately and immersed into 4 ml of 4 °C isolation buffer (mannitol 200 mm, sucrose 50 mM, potassium phosphate 5 mm, EGTA 1 mm, 3-(N-morpholino)propanesulfonic acid 5 mm and bovine serum albumin 0.10% pH 7.2). The tissue was then minced (while in solution) with a pair of scissors. The buffer was tipped to decant the original buffer and 2 ml of 4 °C isolation buffer containing 20% dimethyl sulfoxide was added. Samples were then frozen on dry ice. Samples were coded and stored at −80 °C for later isolation of mitochondria.

### Mitochondrial function analyses

To isolate mitochondria from coded PFC samples, the tissue was rapidly thawed before being homogenized first with a Teflon homogenizer and then with a handheld homogenizer for 2 × 10 s at the lowest-speed setting. Homogenates were then spun in a centrifuge at 800 *g* for 5 min at 4 °C. The supernatants were then spun in a centrifuge at 10 000 *g* for 10 min at 4 °C. The mitochondrial enriched fraction was then re-suspended in mitochondrial assay solution (70 mm sucrose, 220 mm mannitol, 5 mm KH_2_PO_4_, 5 mm MgCl_2_, 2 mm HEPES, 1 mM EGTA, 0.2% fatty acid-free bovine serum albumin), and protein content was determined using the BCA method. Mitochondrial protein (2.5 μg) in 50 μl mitochondrial assay solution was added to 24-well Seahorse V7 plates in triplicate. Mitochondrial function was assessed in mitochondrial assay solution supplemented with 5.5 mm succinate and 2.2 mm rotenone using the Seahorse XF24 Analyzer (Seahorse Bioscience, Shanghai, China) at 37 °C. Multiple cycles of 60-s mix and 4-min measure were used to establish state III and state IV respiration following injection of 2 mm ADP. Respiration rates were determined in point by the point mode.

### Statistical analyses

In all instances, the Shapiro–Wilk test of normality and Levene's test for homogeneity of variance were utilized. Data points more than 2 s.d.'s from the mean were deemed ‘extreme' outliers and were excluded from the analysis. When data were normally distributed, one-way analysis of variance was conducted, where it was followed by Sidak's *post hoc* tests. When these assumptions were violated, the data were analyzed by non-parametric Kruskal–Wallis one-way tests. For experimental questions with only two specific groups of interest, an independent *t*-test was used. Statistical significance was set at *α*=0.05. Analyses were performed using GraphPad Prism 6.0 (GraphPad Software, La Jolla, CA, USA) and IBM SPSS package 22.0 (IBM, Armonk, NY, USA).

## Results

### Validation of antidepressant resistance

The first phase of the experiment (Figure 1a) started with two groups of animals (with no surgery) receiving daily administration for 14 days of either ACTH (100 μg per day, *n*=12) or vehicle (water, *n*=8). These animals were then further randomly allocated into four groups (two under each condition where *n*=4–6) for imipramine (10 μg kg^−1^ per day) or saline treatment in order to measure response to this tricyclic antidepressant.

Antidepressant resistance to imipramine was demonstrated in our ACTH-treated animal model. No differences in locomotor activity were detected between groups, precluding a motor component in the observed effects in the FST (Supplementary Figure S1 and Figure 1).

**Figure 2:**
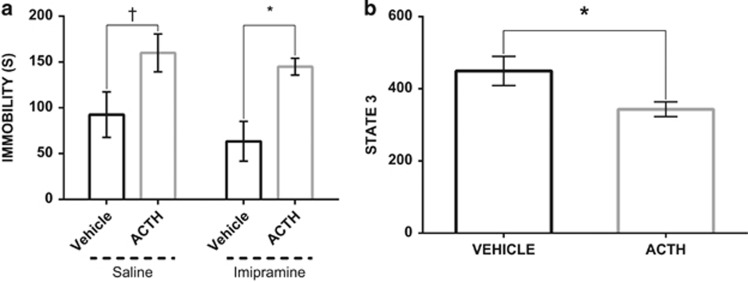
(**a**) Differential effects of imipramine on forced swim test (FST) immobility time between adrenocorticotropic hormone (ACTH)-pretreated- and vehicle animals (*n*=8–12). Saline-treated animals responded to the antidepressant-like effects following imipramine administration. Treatment resistance induced with a chronic treatment of ACTH for 14 days; (**b**) ACTH animals show lower capacity to generate ATP in response to the energy demand relative to the vehicle group. State 3 respiration point represents a maximal ADP stimulation respiration. The values are displayed as means and ±s.e.m. *P<0.05, ^†^*P*=0.07.

With respect to immobility time in the FST, one-way analysis of variance revealed a statistically significant difference between the four treatment conditions (*P*<0.05). ACTH treatment increased immobility time in the FST, and this was not reversed by treatment with imipramine (Figure 2a). Therefore, administration of ACTH induced a model of treatment-resistant depression in these rats.

### Reduced mitochondrial function in ACTH-treated animals

In order to quantify mitochondrial function, respiration analyses were performed on mitochondria isolated from the PFC using a Seahorse XF Analyzer.

State 3 respiration, the peak respiration achieved following addition of excess ADP, is a widely accepted measure of mitochondrial capacity. An unpaired *t*-test revealed a statistically significant difference in state 3 respiration, indicating that ACTH treatment reduced the capacity for ATP production (*P*<0.05; Figure 2b).

### NAc DBS efficacy in imipramine-resistant animals

The second phase of the experiment included animals selected to receive continuous DBS stimulation for 7 days. The SHAM group denotes animals with electrode placement in the NAc with no electrical stimulation, whereas animals within the DBS group had active stimulation to the NAc. The SURG group indicates animals with four jewelry screws placed on the skull cap. CTRL condition refers to animals with no surgical procedures and only receiving the drug (or vehicle) administration. In total, this phase included eight groups, four (DBS, SHAM, SURG and CTRL) of which belonging to either ACTH or vehicle treatment condition (*n*=6–12).

No differences in locomotion during the OFT between groups suggest that there was no motor impairment or damage to confound findings in the FST analyses (*P*>0.05; Supplementary Figure 2).

**Figure 3:**
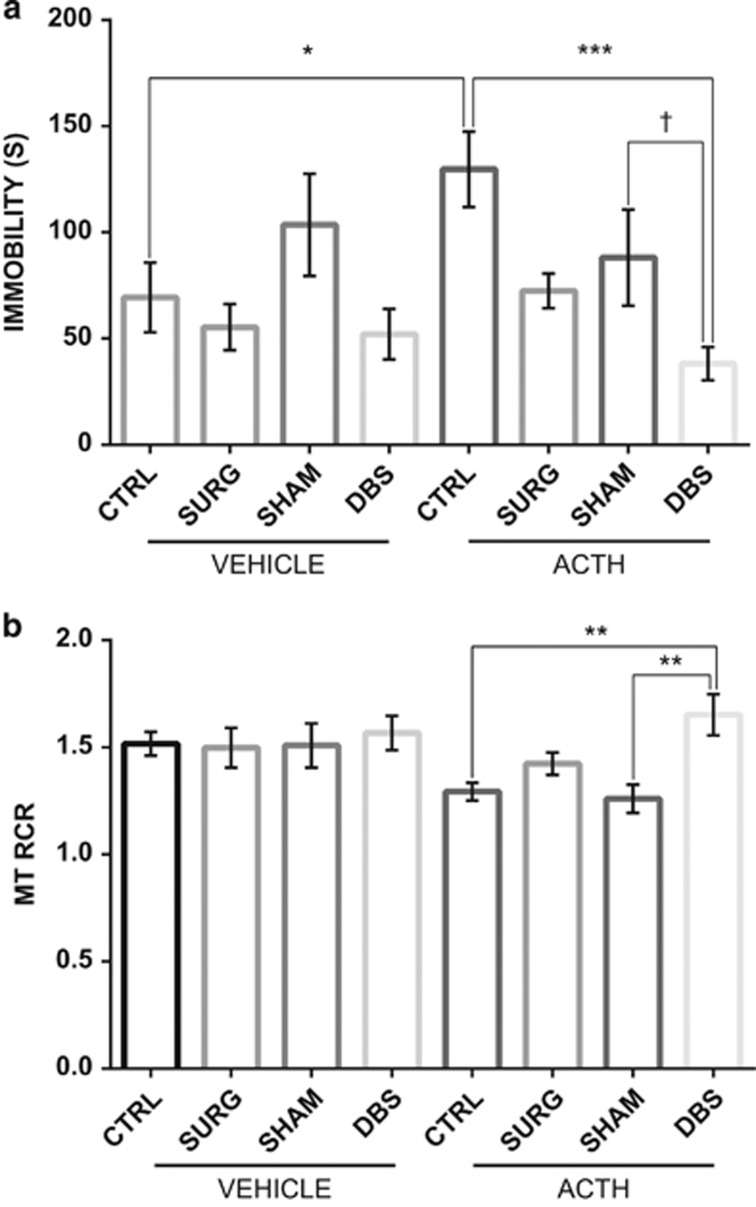
(**a**) Effects of deep-brain stimulation (DBS) on the forced swim test in adrenocorticotropic hormone (ACTH)-treated animals (*n*=7–12). Nucleus accumbens (NAc) DBS significantly decreased time spent in an immobile way in animals previously shown to be non-responsive imipramine. In comparison, DBS did not yield significant antidepressant-like effects on vehicle animals. (**b**) Mitochondrial function in the prefrontal cortex (PFC), represented as the respiratory control ratio (RCR). Animals following NAc DBS show significantly greater mitochondrial RCR relative to ACTH-treated CTRL and SHAM animals. The values are displayed as means and ±s.e.m. *P<0.05, **P<0.01, ***P<0.001, *t*=0.08.

The FST was used to investigate the antidepressant efficacy of NAc DBS after 7 days of uninterrupted stimulation in animals presumed non-responsive to imipramine treatment. When compared with the phase 1 immobility scores of the ACTH group following imipramine administration, there was a 76% reduction in their immobility time after continuous high-frequency stimulation. A one-way analysis of variance revealed a statistically significant difference between ACTH-treated groups in their final FST (*P*<0.05; Figure 3a). In ACTH-treated rats, NAc DBS decreased immobility time in FST by 54% compared with controls (*P*<0.05).

In contrast, saline-treated animals with DBS did not display significant differences in their immobility time compared with that of the saline control group (Figure 3a).

### Mitochondrial function and antidepressant response

Respiratory control ratio (RCR) is the ratio of state 3 to state 4 respiration, and it measures the coupling efficiency of mitochondria. A lower RCR would indicate mitcohondrial dysfuncion that is either impeding ATP production, increasing uncoupled respiration or both. NAc DBS increased RCR in ACTH-treated rats compared with the SHAM and control groups (*P*<0.05; Figure 3b). No differences were seen in the vehicle-treated rats.

### Heightened locomotor activity in ACTH-treated animals following DBS electrode placement

When analyzing OFT data, it was evident that there was a bimodal distribution, with a subset of rats (in the DBS and SHAM groups) exhibiting markedly increased activity levels in terms of distance traveled. We selected this subset based on the distance traveled greater than mean+1 s.d. of the control (vehicle) animals (*M*=29014, SD=5372). Any animals with OFT trial performance beyond this point were considered hyperactive.

**Figure 4:**
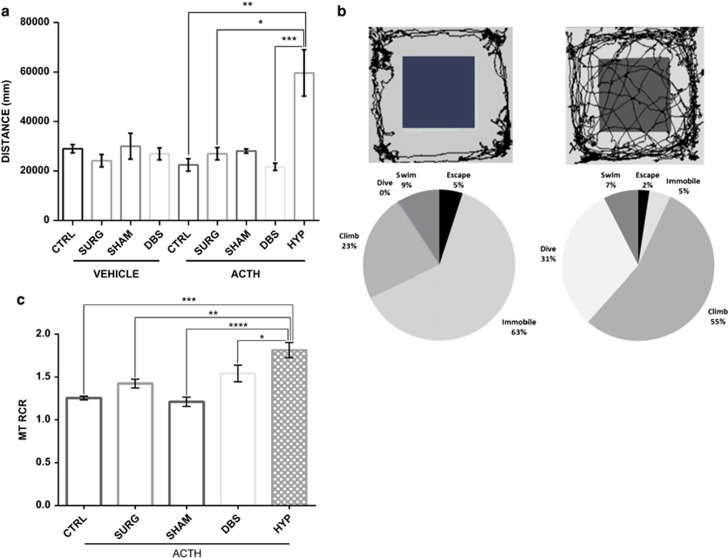
(**a**) A subset of animals developed hyperactive motor activity over the course of adrenocorticotropic hormone (ACTH) treatment (only evident in deep-brain stimulation (DBS) or sham surgery animals with ACTH on board). No such effects were observed in vehicle animals. Animals in this heightened locomotor activity subgroup significantly differed in their distance traveled compared with other ACTH groups. (**b**) Representative trace image of control ACTH versus hyperactive animal and percentage of time engaged in each coping style during forced swim test (FST)—representation of coping behaviors for control ACTH and manic-like phenotype animals during the FST training. Persistent dive, escape and active climbing behaviors indicate that these animals had an exaggerated drive to escape the FST apparatus compared with controls. (**c**) Animals exhibiting mania-like behaviors (HYP) show heightened respiratory control ratio (RCR) relative to other ACTH-pretreated groups. The values are displayed as means and ±s.e.m. *P<0.05, **P<0.01, ***P<0.001, ****P<0.0001.

Given violations to a test of normality (*W*=0.701, *P*<0.05) and tests of homogeneity of variance (*F*_8,71_=4.838, *P*<0.05), a Kruskal–Wallis test was used to confirm a significant group effect where hyperactive animals traveled the furthest during the OFT (*P*<0.05; Figure 4a).

The OFT results, together with representative trace image (Figure 4b), clearly distinguish animals with heightened locomotor behavioral patterns that were followed by extreme application of models of active coping strategies in the FST including persistent diving and escape actions (*P*<0.05, Figure 4b). These behaviors persisted and were recognizable in their home-cage setting (see Supplementary Videos 1 and 2).

Interestingly, when examining the RCR (state 3/state 4), we observed an increase in mitochondrial function in the hyperactive rats relative to other groups pretreated with ACTH (*P*<0.05; Figure 4c).

## Discussion

This study aimed to examine the mechanisms underlying TRD and antidepressant activity in terms of mitochondrial function. First, the data provide evidence for treatment resistance to imipramine, induced by a chronic administration of ACTH. Second, our current findings show reduced mitochondrial capacity in treatment-resistant ACTH-treated animals, which was restored by continuous NAc DBS for 7 days. Concurrently, NAc DBS reduced immobility time in the FST. Lastly, a subgroup of ACTH animals developed a mania-like phenotype, suggested by their adoption of increased behavioral patterns alongside heightened mitochondrial function. Together, these results support a biphasic role for mitochondrial function in regulation of both depressive and manic mood states and additionally reflecting treatment response.

Chronic administration of ACTH interfered with the treatment response to a tricyclic antidepressant and lowered mitochondrial state 3 respiration rate, indicative of reduced ATP production capacity. One possible mechanism whereby ACTH contributes to this dysfunction may be through adaptations of the hypothalamic pituitary adrenal axis, which has consistently been shown to be hyperactive in patients suffering from depression.^32^ Daily administration of ACTH blocks antidepressant efficacy and alters the key PFC monoamine concentration following stress, and downregulates glucocorticoid responses.^19^ Mitochondria participate in stress responses in part by sensing levels of glucocorticoids. Receptors for glucocorticoids exist within the mitochondria, or translocate from the cytoplasm into mitochondria in the presence of their ligand, endowing these organelles with the ability to sense and readily respond during acute stress.^33^

We were secondly able to reverse the imipramine-resistant effects of ACTH using bilateral NAc DBS. The efficacy of NAc DBS in a model of treatment resistance was observed using well-validated behavioral paradigms. These findings corroborate with previous literature on the potential therapeutic effects of NAc DBS for TRD.^27, 29, 34^ Following 1 week of DBS, animals previously resistant to tricyclic antidepressant responded, indicated by reduced utilization of passive coping strategies during the FST. The FST is established as a valid tool for screening antidepressant effects, specifically observing the animals' behavioral despair and helplessness under stress.

Mitochondrial dysfunction can be regarded as the inability of mitochondria to appropriately produce ATP in response to energy demands. ‘Healthy' mitochondria under standard conditions have a high RCR—in other words, a large increase in the respiration rate with ADP administration followed by a return to state 4. Mitochondrial respiratory control encapsulates one of the main functions of mitochondria, which is their ability to idle at a low rate yet respond to an ADP-triggered demand by generating ATP at a high rate. ACTH-treated rats had significantly lower RCR, suggestive of mitochondrial dysfunction. Notably, a high RCR was found in ACTH-pretreated animals following DBS treatment compared with ACTH SHAM and ACTH CTRL, suggesting that mitochondrial efficiency is increased with NAc DBS. As values are substrate- and tissue-dependent, there is no absolute RCR value that is diagnostic of dysfunctional mitochondria. Mitochondrial respiratory control is a complex function whose values respond to numerous factors, and this complexity is its main strength: a change in almost any aspect of oxidative phosphorylation will change RCR.^35^

Additional physiological functions of mitochondria include the generation and detoxification of reactive oxygen species, involvement in apoptosis, regulation of cytoplasmic and mitochondrial matrix calcium, synthesis and catabolism of metabolites and the transport of the organelles themselves to the correct location within the cell.^35^ Notably, many of these functions are altered in mood disorders, including dysregulated intracellular calcium, increased oxidative stress and apoptosis.^36, 37^ From these results, it seems likely that mitochondria have a role in the neuroprogression of major depressive disorder and in antidepressant treatment response.

Increasingly, emerging data show an inextricable interconnection between oxidative stress and inflammatory responses.^38^ A recent meta-analysis indicated globally increased cell-mediated immunity and macrophage activities in major depression.^39^ In light of converging findings, the activation of immunoinflammatory pathways, and oxidative stress coupled with mitochondrial dysfunction, may be strong contributors to the progression and persistence of TRD.

Finally, following NAc DBS, we observed a subgroup of animals with markedly higher locomotor activity, exemplified by increased rates of active climbing and frequent diving. These animals first exhibited such a phenotype with electrode implantation and ACTH administration, which was further exacerbated by active DBS stimulation. Animals not receiving NAc DBS preferentially implemented passive coping strategies in the FST. Mania-like activity included persistent interest in novel stimuli and areas outside their provided enclosure as well as erratic motor movements. In addition, these home-cage behavioral patterns were also evident in their performance during behavioral tests. Notably, a high RCR was found for these animals, offering a possible overcompensatory mechanism where increased mitochondrial function is responsive to the great energy demands. Overall, these observations, together with resistance to imipramine, are indicative of induction of a mania-like phenotype in a vulnerable subset of animals following hypothalamic pituitary adrenal stimulation and a physical disruption to the dopaminergic mesoaccumbens pathway. Future studies will need to further quantify these mania-like characteristics, investigate the fine-grained biological mechanisms mediating such an effect and determine factors predisposing to vulnerability for this behavioral phenotype. However, this paradigm does suggest a putative animal model of mania, with face validity from the perspective of neurocircuitry and neurochemistry.

It is important to emphasize that neither unipolar nor bipolar depression are necessarily classic (or primary) mitochondrial disorders. While noting that some people with primary mitochondrial disorders such as MELAS have very high rates of bipolar-like symptoms,^40^ by and large those with bipolar disorder do not exhibit the symptoms of classic mitochondrial disorders. Instead, emerging data suggest that upstream abnormalities (likely encoded in the nucleus) converge at mitochondrial function,^38^ leading to altered synaptic plasticity and impaired cellular resilience.^41^ Given the biphasic nature of bipolar disorder, it is hypothesized that the disorder represents a bi-directional state-dependent alteration in mitochondrial regulation.^42^ Our data presented here suggest that it may be possible to develop a new model of bipolar disorder with a biphasic mitochondrial and behavioral response to ACTH administration.

The results of this study should be considered in light of limitations shared by many translational models of psychiatric disorders. First, the models such as the FST alone may not be sufficient to provide strong predictive validity of antidepressant-like effects of DBS. Second, animals do not have genotype/phenotype features that may resemble all clinical depression-like and bipolar states before testing, apart from their resistance to imipramine and heightened locomotor activities. Despite these aforementioned caveats, preclinical models are integral for the study of DBS efficacy, and offer mechanistic contributions to our understanding in the field not available in human studies. In humans, the appraisal of clinical effects of DBS to date is primarily carried out through the use of subjective measures. Hence, placebo/sham effects are difficult to incorporate and measure in clinical trials. Preclinical research can help characterize the biological mechanisms of DBS and DBS-induced mania, as well as to further isolate the unique effects of chronic stimulation in antidepressant mechanisms.

All in all, mitochondrial dysfunction seems to have a role in the impairments of cellular plasticity and resilience manifested in the context of mood-related disorders. DBS of the NAc was effective in reducing immobility time in antidepressant-resistant animals. Bearing in mind that PFC regions heavily project to the accumbens and some of the projections from the PFC to subcortical areas run through white matter fibers of the frontal cortex, these findings suggest that mitochondrial function within the PFC may have an impact on motivated behaviors associated with NAc involvement. Possible factors that may underlie the altered bioenergetic phenotype observed in this preclinical model of tricyclic antidepressant resistance include the following: dopamine dysregulation, mitochondrial dysfunction, oxidative stress, inflammation and glial cell abnormalities.

In the future, the field of neuromodulation must look to basic research to establish the key factors influencing different subpopulations, driving mood states and affecting treatment responsivity. The challenges of developing an animal model of treatment-resistant depression and/or mania are many; however, the potential benefits of such a feat are no doubt worthwhile to help unlock the neurobiology of these illness states.
