# Supplementary material for: Nucleus accumbens deep-brain stimulation efficacy in ACTH-pretreated rats: alterations in mitochondrial function relate to antidepressant-like effects
Source: Transl Psychiatry. 2016 Jun 21;6(6):e842–. doi: 10.1038/tp.2016.84 (PMC4931612; doi:10.1038/tp.2016.84)
Supplement: Supplementary Video Legends [file tp201684x3.docx]

**Supplementary Video Legends**

Video 1: Typical day time home cage behavior of a control ACTH-treated rat.

Video 2: Typical day time home cage behavior of an ACTH-treated rat with NAc DBS electrode displaying the hyperactive mania-like phenotype.
