# Supplementary figures and images for: Nucleus accumbens deep-brain stimulation efficacy in ACTH-pretreated rats: alterations in mitochondrial function relate to antidepressant-like effects
Source: Transl Psychiatry. 2016 Jun 21;6(6):e842–. doi: 10.1038/tp.2016.84 (PMC4931612; doi:10.1038/tp.2016.84)

S1

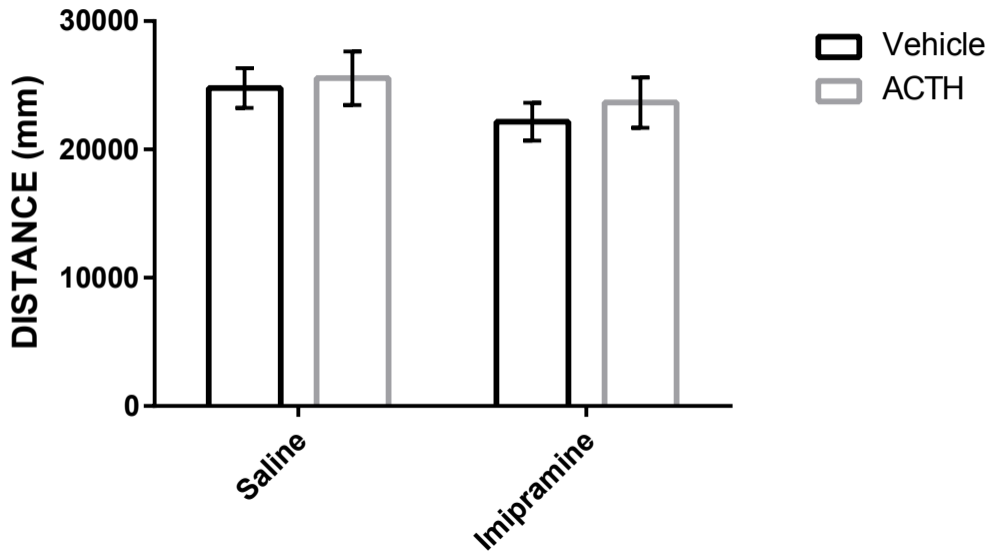

Supplement: Supplementary Figure 1 [file tp201684x4.pdf]

**S2**

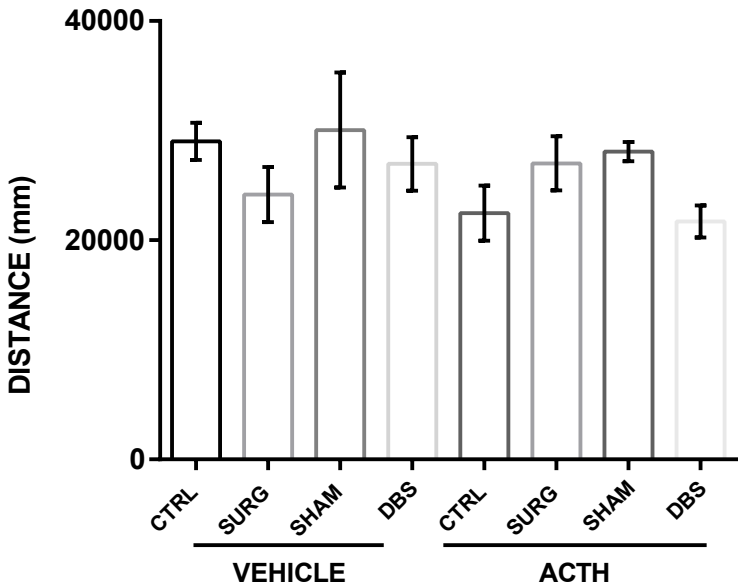

Supplement: Supplementary Figure 2 [file tp201684x5.pdf]
